# Shift in prevalence and systemic inflammation levels from NAFLD to MAFLD: a population-based cross-sectional study

**DOI:** 10.1186/s12944-023-01947-4

**Published:** 2023-10-28

**Authors:** Qingdan Liu, Meilan Han, Meilan Li, Xiaoyin Huang, Ruimei Feng, Wanxin Li, Jun Chen, Haiying He, Wenxin Zheng, Zhijian Hu, Shanshan Du, Weimin Ye

**Affiliations:** 1 Department of Epidemiology and Health Statistics, School of Public Health, Fujian Medical University, University Town, No 1, Xue Yuan Road, Fuzhou, 350108 Fujian China; 2 Department of Ultrasonography, Fuqing Hospital, Fuqing, China; 3 Infection Control Department, The Fifth Hospital of Fuqing City, Fuqing, China; 4 Department of Medical Epidemiology and Biostatistics, Karolinska Institutet, Stockholm, Sweden

**Keywords:** MAFLD, NAFLD, Prevalence, Systemic inflammatory status, General population

## Abstract

**Background:**

Variations in the prevalence and systemic inflammatory (SI) status between non-alcoholic fatty liver disease (NAFLD) and newly defined metabolic dysfunction-associated fatty liver disease (MAFLD) have only been reported by few studies. Hence, this study aimed to compile data on the prevalence and the systemic inflammation levels of MAFLD and NAFLD in a general population from Southeast China was summarized to explore the potential effect of the transformation of disease definition.

**Methods:**

A total of 6718 general population participants aged 35–75 were enrolled. Logistic regression and restricted cubic spline (RCS) models were used to examine the relationship between 15 SI indicators and NAFLD and MAFLD. The predicted values of MAFLD and NAFLD were analyzed using the receiver operating characteristic (ROC) curve.

**Results:**

The prevalence of MAFLD and NAFLD was 34.7% and 32.4%, respectively. Their overlapping rate was 89.7%, while only 8.3% and 1.9% of participants were MAFLD-only and NAFLD-only. Among three FLD groups, the MAFLD-only group had the highest levels of 8 SI indicators, including CRP, WBC, LYMPH, NEUT, MONO, ALB, NLR, and SIRI. The non-FLD group had the lower levels of all 15 SI indicators compared with all FLD subgroups. The odds ratios (ORs) of 10 SI indicators were significant in both multivariable-adjusted logistic regression and RCS analyses of MAFLD or NAFLD, including CRP, WBC, LYMPH, NEUT, MONO, ALB, PLR, LMR, ALI and CA. ROC analysis showed that the AUC values of all SI were lower than 0.7 in both MAFLD and NAFLD.

**Conclusions:**

MAFLD could cover more FLD than NAFLD, and the MAFLD-only group had a more severe inflammation status, whereas the NAFLD-only exhibited lower levels. Moreover, there was not a high AUC and a high sensitivity of SI indicators, suggesting that SI indicators are not good indicators to diagnose NAFLD/MAFLD.

**Supplementary Information:**

The online version contains supplementary material available at 10.1186/s12944-023-01947-4.

## Background

Nonalcoholic fatty liver disease (NAFLD) is the most prevalent liver disease globally, with an estimated prevalence of 25% [1, 2]. It is strongly associated with a range of metabolic disorders, including hyperglycemia, hypertension, abdominal obesity, and dyslipidemia [3]. In 2020, a recommendation was to introduce metabolic dysfunction-associated fatty liver disease (MAFLD) [4], This change in terminology aimed to shift the focus away from alcohol consumption as the defining factor in NAFLD, emphasizing instead the role of metabolic disorders in the progression of NAFLD-related pathologies [5]. In 2021, two separate meta-analyses highlighted a significant difference in the prevalence rates of MAFLD and NAFLD. Specifically, MAFLD identified a larger number of patients, though it is important to note that there was still a considerable overlap between the two conditions [6, 7]. It is crucial to recognize that the prevalence of subgroups such as MAFLD-only, NAFLD-only, and overlap-FLD can vary significantly based on the proportions of metabolic abnormalities and other concurrent conditions, as defined by their respective criteria. [6]. In China, the reported prevalence of MAFLD ranging from 21.0% to 46.7% [8–14] and prevalence of NAFLD ranging from 29.3% to 32.9% [15–17], respectively it is varied largely among geographic regions. Thus, more studies among general populations are warranted to understand the similarities and differences between the two conditions, before the transformation from NAFLD to MAFLD.

Inflammation is a physiological response to tissue injury or infection, leading to the release of various inflammatory mediators. When inflammation persists over time, it can lead to chronic systemic inflammatory changes, which can worsen tissue damage [18, 19]. The status of systemic inflammation (SI) is widely acknowledged as a primary pathogenic factor in the advancement of steatohepatitis, fibrosis, and adverse outcomes associated with chronic liver diseases [20–23]. In the meantime, studies have reported that MAFLD tends to be associated with advanced liver disease compared to NAFLD [6]. The potential disparity in systemic inflammation (SI) levels between MAFLD and NAFLD is still uncertain, yet it could bear significant implications for the shift in diagnostic criteria. This study seeks to examine the association between MAFLD and NAFLD from an inflammatory standpoint, and to determine whether the distinct definitions of MAFLD and NAFLD result in differences in the affected populations. Therefore, the current study sought to investigate and compare the prevalence of MAFLD and NAFLD in Southern China, and explore the chronic inflammatory status and indicators of MAFLD, NAFLD, and three subgroups, including MAFLD-only, NAFLD-only, and overlap-FLD. The results may provide insight into the clinical relevance of the novel MAFLD definition from the SI perspective.

## Methods

### Study design and population

**Fig. 1:**
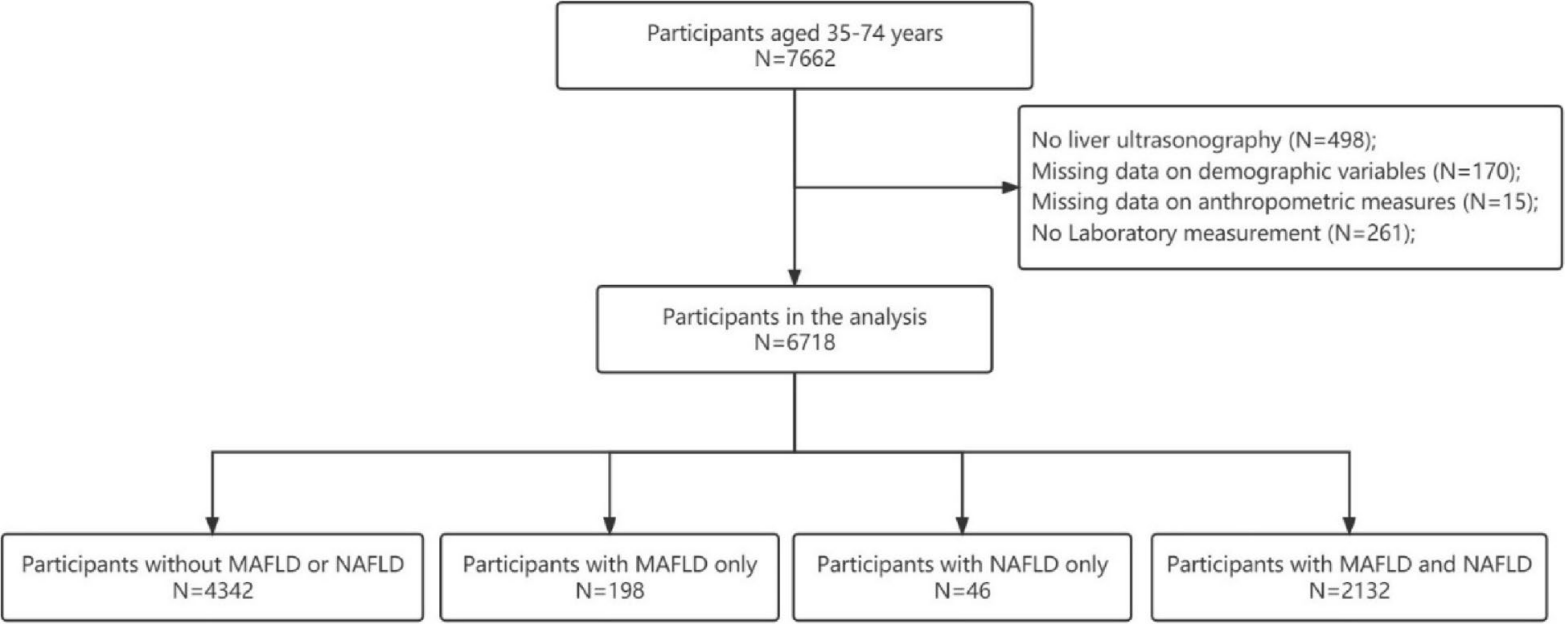
Flowchart showing selection of study population

This study was conducted in Fuqing, Fujian Province in Southern China from July 2020 to June 2021, which recruited residents aged 35–75 years. Of the 7662 participants, 7164 underwent liver ultrasound examination. Participants with missing anthropometric results or important clinical and laboratory data were excluded from the analysis. A total of 6718 individuals were included in the final analysis. A flowchart of the participant enrollment process is shown in Fig. 1.

The study protocol was approved by the ethical committee of the Fujian Medical University (approval number: 2020–58), and written informed consent was obtained from all participants.

### Data collection

Data were gathered by well-trained interviewers and examiners, including demographic variables, anthropometric measures, laboratory measurements, and liver ultrasonography.

#### Demographic variables

A face-to-face interview was conducted using a structured electronic questionnaire by well-trained interviewers, to collect participants’ information, including socio-demographic characteristics, lifestyle variables (smoking, alcohol drinking, and physical activity), and history of disease and medication. The electronic questionnaire was independently developed by the research group (https://cohort.fjmu.edu.cn/cobl). The interview process was tape-recorded, and the degree of cooperation of the respondents and the reliability of the questionnaire were evaluated.

#### Anthropometric measures

Anthropometric measurements, including height, body weight, waist circumference (WC), hip circumference (HC), and systolic and diastolic blood pressure (SBP and DBP) were measured by trained staff. The body mass index (BMI) was also calculated as body weight in kilograms divided by height squared in meters (kg/m^2^). WC and HC were measured with a tape measure to horizontally circle the waist and hips of all subjects, after they took off their coats, loosened their belts, stood naturally on both legs, and maintained calm breathing. Blood pressure (BP) measurements were taken on the right upper arm by trained employees using an electronic BP monitor (OMRON, Kyoto, Japan) at the heart level. The BP was measured twice with an interval of 30 s. When the difference between two SBP/DBP measurements was greater than 5 mmHg, a third measurement was taken. The two closest of all measurements were taken to calculate the average SBP and DBP, which were used in the analysis.

#### Laboratory measurement

Fasting blood was collected from all participants after fasting for at least 8 h. All participants without self-reported diabetes mellitus were invited to perform a 75 g oral glucose tolerance test (OGTT), and 2-h post-load blood samples were collected. Fasting blood glucose (FBG), 2-h post-load blood glucose (2 h-PG), triglyceride (TG), high-density lipoprotein cholesterol (HDL-c), albumin (ALB), C-reactive protein (CRP), alanine aminotransferase (ALT), and aspartate aminotransferase (AST) were measured using an automatic analyzer (Toshiba, Tokyo, Japan). Glycosylated hemoglobin (HbA1c) was measured using a high-performance liquid chromatography method (ARKRAY, Osaka, Japan). Fasting insulin (FINs) was measured by electrochemiluminescence immunoassay (Roche Diagnostics, Munich, Germany). Homeostasis model assessment of insulin resistance (HOMA-IR) was calculated as follows: FINs × FBG/22.5 [24]. White blood cell (WBC), lymphocyte (LYMPH), neutrophils (NEUT), monocyte (MONO), and platelet (PLT), and the mean platelet volume (MPV) were measured using an automated hematology analyzer (SYSMEX, Kyoto, Japan).

#### Liver ultrasonography

Abdominal ultrasound was performed after overnight fasting and was completed by well-trained technicians using a portable full-digital color Doppler ultrasound diagnostic instrument (Hitachi, Tokyo, Japan).

### Diagnostic criteria of NAFLD, MAFLD, and metabolic disorders

NAFLD was defined as evidence of hepatic steatosis based on abdominal ultrasound and the exclusion of significant alcohol consumption (defined as ≥ 30 g/day for men and 20 g/day for women, respectively) [25].

MAFLD was defined as evidence of fatty liver based on abdominal ultrasound with at least one of the following three conditions [4]: (1) overweight or obesity (BMI ≥ 23.0 kg/m^2^ in Asians); (2) type 2 diabetes mellitus (T2DM); and (3) metabolic dysregulation among non-overweight individuals (BMI < 23.0 kg/m^2^ in Asians).

Hypertension was defined as an average SBP ≥ 140 mmHg, DBP ≥ 90 mmHg, self-reported history of hypertension, and/or taking antihypertensive drugs [26].

T2DM was defined as an FBG ≥ 7.0 mmol/L, 2 h-PG ≥ 11.1 mmol/L, HbA1c ≥ 6.5%, self-reported history of DM, and/or use of antidiabetic drugs [27].

Prediabetes was defined as non-diabetic individuals with an FBG level of 5.6-6.9 mmol/L, 2h-PG of 7.8-11.1 mmol/L, and/or HbA1c of 5.7-6.4% [27].

Hyperlipidemia was defined as triacylglycerols ≥ 2.26 mmol/L and/or total cholesterol ≥ 6.22 mmol/L and/or HDL-c < 1.04 mmol/L and/or LDL-c ≥ 4.14 mmol/L and/or self-reported medication for hyperlipidemia.

### Indicators of the SI level

A total of 15 indicators were applied to evaluate the SI level of the population. CRP, WBC, LYMPH, NEUT, MONO, MPV, and ALB were obtained from laboratory measurements, and eight indicators were calculated according to the following equations: neutrophil-to-lymphocyte ratio (NLR) = NEUT/lymphocyte (LYMPH) [28], derived NLR (d_NLR) = NEUT/(WBC-NEUT) [28], platelet-to-lymphocyte ratio (PLR) = PLT/LYMPH [28], lymphocyte-to-monocyte ratio (LMR) = LYMPH/MONO [28], systemic immune-inflammation index (SII) = PLT × NEUT/LYMPH [29], C-reactive protein-to-albumin ratio (CA) = CRP/ALB [30], advanced lung cancer inflammation index (ALI) = BMI × ALB/NLR [31], and systemic inflammation response index (SIRI) = NEUT × MONO/LYMPH [32].

### Statistical analysis

Statistical analysis was performed using SAS (version 9.4, America), and a two-tailed* P*-value of < 0.05 was considered statistically significant. Continuous variables were presented as mean ± standard deviation (SD) or median (interquartile range (IQR)) based on data distribution and were compared using independent Student’s t-test or one-way analysis of variance (ANOVA). The Fisher’s least significant difference (LSD) method was used for pairwise comparison between groups. Categorical variables were expressed as numbers and percentages and compared using the Chi-squared test. Non-normally distributed data were analyzed using the nonparametric test and logarithmically transformed to normality when appropriate.

Multivariable logistic regression models were applied to calculate the odds ratios (ORs) and corresponding 95% confidence intervals (CIs) for NAFLD and MAFLD with different inflammatory indicators. To prevent the bias caused by any possible leverage value, restricted cubic spline (RCS) models were used to fit the non-linear relationship between inflammatory status indicators and MAFLD and NAFLD. Additionally, to analyze the predictive power of 15 inflammatory indicators for MAFLD and NAFLD and determine the best threshold for each parameter, the receiver operating characteristic (ROC) curve was used to analyze each parameter and find the point at which the sum of sensitivity and specificity was maximized to determine the best threshold for each parameter.

## Results

### Prevalence and characteristics of MAFLD, NAFLD, and their subgroups

The demographics, anthropometrics, and laboratory test characteristics of 6718 subjects are presented in Table 1. The median age of the participants was 57 years (range, 50–65 years). Out of all the participants, 34.7% were male. A total of 2330 individuals were diagnosed with MAFLD, yielding a prevalence rate of 34.7%. Within the MAFLD group, the percentages of elderly individuals, unemployed individuals, farmers, as well as those with hypertension and T2DM were higher compared to the non-MAFLD group (all* P* < 0.05). Compared with the non-MAFLD group, the MAFLD group had dramatically higher levels of WC, BMI, SBP, DBP, ALT, AST, TG, FBG, and 2 h-PG and significantly lower HDL-c levels.

**Table 1:** General characteristics of subjects

	Overall (*n* = 6718)	non-MAFLD (*n* = 4388)	MAFLD (*n* = 2330)	non-NAFLD (*n* = 4540)	NAFLD (*n* = 2178)
Age, years	57.0 (50.0, 65.0)	57.0 (49.0, 65.0)	58.0 (52.0, 66.0)*	57.0 (49.0, 65.0)	58.0 (52.0, 66.0)*
Sex					*
Male	2328 (34.7)	1500 (34.2)	828 (35.6)	1657 (36.5)	671 (30.8)
Female	4390 (65.4)	2889 (65.8)	1501 (64.5)	2883 (63.5)	1507 (69.2)
Education level			*		*
Less than primary school	2268 (33.8)	1441 (32.8)	827 (35.5)	1444 (31.8)	824 (37.8)
Primary school	2292 (34.1)	1514 (34.5)	778 (33.4)	1567 (34.5)	725 (33.3)
Middle school	1555 (23.2)	1049 (23.9)	506 (21.7)	1107 (24.4)	448 (20.6)
High school or higher	603 (9.0)	385 (8.8)	218 (9.4)	422 (9.3)	181 (8.3)
Occupation			*		*
Farmer or unemployment	4878 (72.6)	3208 (71.9)	1754 (73.8)	3233 (71.2)	1645 (75.5)
Worker	690 (10.3)	502 (11.3)	202 (8.5)	514 (11.3)	176 (8.1)
Sales or service	417 (6.2)	282 (6.3)	141 (5.9)	293 (6.5)	124 (5.7)
Official job	663 (9.9)	428 (9.6)	251 (10.6)	451 (9.9)	212 (9.7)
Other	70 (1.0)	43 (1.0)	28 (1.2)	49 (1.1)	21 (1.0)
Smoking history					*
Never-smoker	4957 (73.8)	3246 (74.0)	1711 (73.5)	3261 (71.8)	1696 (77.9)
Former smoker	598 (8.9)	373 (8.5)	225 (9.7)	410 (9.0)	188 (8.6)
Current smoker	1163 (17.3)	770 (17.5)	393 (16.9)	869 (19.1)	294 (13.5)
T2DM			*		*
No	5430 (80.8)	3865 (88.1)	1565 (67.2)	3967 (87.4)	1463 (67.2)
Yes	1288 (19.2)	524 (11.9)	764 (32.8)	573 (12.6)	715 (32.8)
Hypertension			*		*
No	3584 (53.4)	2695 (61.4)	889 (38.2)	2720 (59.9)	864 (39.7)
Yes	3134 (46.7)	1694 (38.6)	1440 (61.8)	1820 (40.1)	1314 (60.3)
BMI, kg/m^2^			*		*
< 18.5	163 (2.4)	162 (3.7)	1 (0)	161 (3.6)	2 (0.1)
18.5–23.9	3265 (48.6)	2848 (64.9)	417 (17.9)	2837 (62.4)	428 (19.7)
24.0–27.9	2518 (37.5)	1216 (27.7)	1302 (55.9)	1330 (29.3)	1188 (54.5)
≥ 28.0	772 (11.5)	162 (3.7)	610 (26.2)	212 (4.7)	560 (25.7)
WC, cm	82.8 (76.4, 89.2)	79.4 (73.8, 85.1)	89.2 (84.0, 94.8)*	80 (74, 86)	88.8 (83.4, 94.2)*
Hipline, cm	93.8 (89.8, 98.0)	92.0 (88.4, 95.8)	97.2 (93.5, 101.2)*	92.2 (88.6, 96.0)	97.0 (93.2, 101.0)*
SBP, mmHg	131.5 (119.0, 146.5)	128.0 (116.5, 142.5)	138.0 (125.0, 152.0)*	128.5 (117.0, 143.0)	138.0 (124.5, 152.0)*
DBP, mmHg	84.0 (7.07, 91.5)	82.0 (75.5, 89.5)	88.0 (81.0, 95.0)*	82.5 (75.5, 90.0)	87.5 (80.5, 94.5)*
TG, mmol/L	1.1 (0.8, 1.6)	1.0 (0.7, 1.3)	1.5 (1.1, 2.1)*	1.0 (0.8, 1.3)	1.5 (1.1, 2.0)*
HDL-c, mmol/L	1.6 (1.4, 1.8)	1.7 (1.4, 1.9)	1.5 (1.3, 1.7)*	1.6 (1.4, 1.9)	1.5 (1.3, 1.7)*
FBG, mmol/L	5.0 (4.6, 5.5)	4.8 (4.5, 5.3)	5.2 (4.8, 6.1)*	4.9 (4.5, 5.3)	5.2 (4.8, 6.1)*
2 h PG, mmol/L	7.3 (6.0, 9.0)	6.9 (5.8, 8.4)	8.2 (6.7, 10.4)*	6.9 (5.8, 8.4)	8.2 (6.7, 10.4)*
FINs	7.1 (4.9, 10.3)	5.9 (4.2, 8.3)	10.1 (7.2, 13.6)*	6.0 (4.3, 8.4)	10.0 (7.2, 13.4)*
ALT, U/L	19.0 (15.0, 26.0)	18.0 (14.0, 23.0)	23.0 (18.0, 31.0)*	18.0 (14.0, 24.0)	23.0 (18.0, 31.0)*
AST, U/L	22.0 (18.0, 26.0)	21.0 (18.0, 25.0)	22.0 (19.0, 27.0)*	22.0 (18.0, 26.0)	22.0 (19.0, 26.0)

Data are presented as median with the interquartile range [M (P25-P75)], or frequency (percentage) [n (%)]

*MAFLD* metabolic associated fatty liver disease, *NAFLD* nonalcoholic fatty liver disease, *T2DM* type 2 diabetes mellitus, *WC* waist circumference, *BMI* body mass index, *SBP* systolic blood pressure, *DBP* diastolic pressure, *TG* triglyceride, *HDL-C* high-density lipoprotein cholesterol, *FBG* fasting blood glucose, *2 h PG* 2-h post-load blood glucose, *FINs* Fasting insulin, *ALT* alanine aminotransferase, *AST* aspartate aminotransferase

^*^P < 0.05 between the non-MAFLD and MAFLD, and non-NAFLD and NAFLD calculated by Wilcoxon Signed Rank Test or Chi-squared test

The prevalence of NAFLD was 32.4%. The proportions of elderly, women, unemployed, farmers, smokers, hypertensive and T2DM patients among NAFLD group were higher than those among non-NAFLD group (all *P* < 0.05). The NAFLD group had dramatically higher levels of WC, BMI, SBP, DBP, ALT, TG, FBG, and 2 h-PG and significantly lower HDL-c levels compared with the non-NAFLD group.

The entire population was regrouped into non-FLD, overlap-FLD, MAFLD-only, and NAFLD-only groups. Participants who met the criteria for both MAFLD and NAFLD were categorized in the overlap-FLD group. The overlapping population included 2132 subjects, with an overlapping rate of 89.7%. Participants who met the inclusion criteria for MAFLD but not NAFLD were classified as MAFLD-only, and those who met the criteria for NAFLD but not MAFLD were considered to be NAFLD-only. The prevalence of MAFLD-only and NAFLD-only was 8.3%, and 1.9%, respectively.

### SI levels of MAFLD, NAFLD, and their subgroups

The 15 SI indicators are shown in Table 2. Except for MPV, d_NLR, and SII, other indicators dramatically differed between MAFLD and non-MAFLD groups, and the MAFLD group had higher levels of SI. Similarly, compared with the non-NAFLD group, NAFLD participants had higher levels of all the indicators, except for MPV, SII, and SIRI.

**Table 2:** The levels of systemic inflammatory indicators in general population, MAFLD and NAFLD

Indicators	Overall (*n* = 6718)	Non-MAFLD (*n* = 4388)	MAFLD (*n* = 2330)	Non-MAFLD (*n* = 4540)	NAFLD (*n* = 2178)
CRP, mg/L	1.11 (0.73, 2.04)	1.00 (0.68, 1.74)	1.39 (0.87, 2.50)*	1.01 (0.69, 1.78)	1.37 (0.87, 2.49)*
WBC, 10^9/L	5.74 (4.90, 6.78)	5.52 (4.72, 6.55)	6.11 (5.32, 7.11)*	5.55 (4.74, 6.58)	6.09 (5.28, 7.10)*
LYMPH, 10^9/L	2.01 (1.65, 2.43)	1.92 (1.58, 2.32)	2.17 (1.81, 2.60)*	1.93 (1.59, 2.34)	2.17 (1.82, 2.60)*
NEUT, 10^9/L	3.17 (2.56, 3.96)	3.05 (2.47, 3.83)	3.36 (2.78, 4.14)*	3.08 (2.48, 3.87)	3.34 (2.75, 4.11)*
MONO, 10^9/L	0.34 (0.27, 0.41)	0.33 (0.27, 0.40)	0.35 (0.29, 0.43)*	0.33 (0.27, 0.41)	0.35 (0.29, 0.42)*
MPV, fL	10.60 (10.00, 11.20)	10.60 (10.00, 11.20)	10.50 (10.00, 11.10)	10.60 (10.00, 11.20)	10.55 (10.00, 11.20)
ALB, g/L	48.80 (47.00, 50.60)	48.70 (46.90, 50.50)	49.00 (47.30, 50.80)*	48.70 (46.90, 50.50)	48.90 (47.30, 50.70)*
NLR	1.57 (1.22, 2.04)	1.60 (1.22, 2.07)	1.53 (1.22, 1.96)*	1.61 (1.23, 2.07)	1.53 (1.20, 1.95)*
d_NLR	1.27 (1.00, 1.61)	1.28 (1.00, 1.62)	1.24 (1.00, 1.57)	1.28 (1.00, 1.62)	1.24 (0.99, 1.57)*
PLR	121.43 (97.95, 150.53)	125.46 (100.00, 156.1)	114.98 (93.91, 142.00)*	125.00 (99.50, 155.32)	116.07 (95.06, 142.04)*
SII	382.53 (284.17, 521.14)	380.66 (278.16, 524.12)	386.62 (293.20, 517.52)	380.82 (279.34, 525.03)	386.67 (292.62, 516.23)
SIRI	0.53 (0.37, 0.75)	0.52 (0.36, 0.75)	0.55 (0.39, 0.76)*	0.53 (0.37, 0.76)	0.54 (0.39, 0.75)
LMR	5.99 (4.78, 7.43)	5.88 (4.68, 7.31)	6.21 (5.05, 7.57)*	5.86 (4.66, 7.29)	6.27 (5.11, 7.67)*
ALI	740.39 (562.69, 974.88)	696.1 (528.42, 915.45)	839.78 (646.5, 1072.40)*	700.82 (530.62, 920.56)	839.05 (646.82, 1074.66)*
CA	0.02 (0.01, 0.04)	0.02 (0.01, 0.04)	0.03 (0.02, 0.05)*	0.02 (0.01, 0.04)	0.03 (0.02, 0.05)*

Data are presented as median with the interquartile range [M (P25-P75)]

*CRP* C-reactive protein, *WBC* white blood cell, *LYMPH* lymphocyte, *NEUT* neutrophils, *MONO* monocyte, *MPV* mean platelet volume, *ALB* albumin, *NLR* neutrophils-to-lymphocyte ratio, *SII* systemic immune inflammation index, *SIRI* systemic immune inflammation response index

^*^P < 0.05 for the comparison of individuals with and without NAFLD or MAFLD by Wilcoxon Signed Rank test

Compared among non-FLD, overlap-FLD, MAFLD-only, and NAFLD-only groups, the MAFLD-only group had the highest levels of CRP, WBC, LYMPH, NEUT, MONO, ALB, NLR, and SIRI, whereas the NAFLD-only group had the highest levels of PLR and the overlap-FLD group had the highest levels of LMR and ALI. The non-FLD group had the lowest levels of all 15 SI indicators (Table 3).

**Table 3:** The levels of systemic inflammatory indicators of MAFLD-only, NAFLD-only, and overlap- MAFLD/NAFLD

Indicators	Non-MAFLD/NAFLD (*n* = 4342)	MAFLD-only (*n* = 198)	NAFLD-only (*n* = 46)	overlap-MAFLD/NAFLD (*n* = 2132)
Age, years	57.0 (49.0, 65.0)	56.0 (48.0, 65.0)	52.0 (46.0, 57.0)	59.0 (52.0, 66.0) *
Sex				*
Male	1477 (34.0)	180 (90.9)	23 (50.0)	648 (30.4)
Female	2865 (66.0)	18 (9.1)	23 (50.0)	1484 (69.6)
T2DM				*
No	3819 (88.0)	148 (74.8)	46 (100.0)	1417 (66.5)
Yes	523 (12.0)	50 (25.2)	0 (0.0)	715 (33.5)
Hypertension				*
No	2652 (61.1)	68 (34.3)	42 (91.3)	822 (38.6)
Yes	1690 (38.9)	130 (65.7)	4 (8.7)	1310 (61.4)
Hyperlipidaemia				*
No	3004 (69.2)	102 (51.5)	35 (76.1)	1082 (50.8)
Yes	1338 (30.8)	96 (48.5)	11 (23.9)	1050 (49.3)
BMI, kg/m^2^				*
< 18.5	160 (3.7)	1 (0.5)	2 (4.3)	0 (0.0)
18.5–23.9	2804 (64.6)	33 (16.7)	44 (95.7)	384 (18)
24.0–27.9	1216 (28)	114 (57.6)	0 (0.0)	1188 (55.7)
≥ 28.0	162 (3.7)	50 (25.2)	0 (0.0)	560 (26.3)
Central obesity				*
No	2884 (66.4)	56 (28.3)	46 (100.0)	451 (21.2)
Yes	1458 (33.6)	142 (71.7)	0 (0.0)	1681 (78.8)
WC, cm	79.5 (73.8, 85.2)	92.7 (88.3, 98.0)	76.45 (74.8, 81.2)	89 (83.6, 94.3) *
Hipline, cm	92.0 (88.4, 96.0)	98.0 (94.0, 101.5)	91.0 (89.0, 92.5)	97.1 (93.5, 101.2) *
SBP, mmHg	128.5 (116.5, 143.0)	137.5 (125.0, 151.5)	116.3 (109.5, 124.0)	138.3 (125.0, 152.5) *
DBP, mmHg	82.0 (75.5, 89.5)	90.5 (83.0, 98.5)	78.3 (75.5, 83.0)	88.0 (81.0, 94.5) *
TG, mmol/L	1.0 (0.74, 1.29)	1.6 (1.2, 2.2)	1.1 (0.9, 1.4)	1.5 (1.1, 2.0) *
HDL-c, mmol/L	1.7 (1.4, 1.9)	1.4 (1.2, 1.6)	1.5 (1.4, 1.7)	1.5 (1.3, 1.7) *
FBG, mmol/L	4.9 (4.5, 5.3)	5.2 (4.7, 6.0)	4.6 (4.5, 4.9)	5.2 (4.8, 6.1) *
2 h PG, mmol/L	6.9 (5.8, 8.4)	7.7 (6.6, 9.7)	6.3 (5.3, 7.3)	8.3 (6.7, 10.5) *
FINs	5.9 (4.2, 8.2)	9.3 (6.7, 13.9)	6.3 (3.9, 8.3)	10.1 (7.3, 13.5) *
ALT, U/L	18.0 (14.0, 23.0)	25.0 (20.0, 38.0)	19.5 (15.0, 26.0)	23.0 (18.0, 31.0) *
AST, U/L	21.0 (18.0, 26.0)	23.0 (19.0, 28.0)	22.0 (17.0, 24.0)	22.0 (19.0, 26.0) *
Alcohol intake, g/d	37.2 (15.1, 96.5)	86.1 (45.2, 167.3)	15.1 (15.1, 15.1)	5.1 (2.6, 14.7) *
Indicators	Non-FLD (*n* = 4342)	MAFLD-only (*n* = 198)	NAFLD-only (*n* = 46)	overlap-FLD (*n* = 2132)
CRP, mg/L	1.00 (0.68, 1.75)	1.43 (0.89, 2.43)	1.10 (0.69, 1.51)	1.39 (0.87, 2.51) *
WBC, 10^9/L	5.52 (4.72, 6.55)	6.23 (5.42, 7.25)	5.30 (4.80, 6.55)	6.11 (5.30, 7.11) *
LYMPH, 10^9/L	1.92 (1.58, 2.32)	2.18 (1.74, 2.55)	1.99 (1.71, 2.25)	2.17 (1.82, 2.61) *
NEUT, 10^9/L	3.05 (2.46, 3.84)	3.47 (2.93, 4.28)	2.98 (2.53, 3.70)	3.36 (2.76, 4.13) *
MONO, 10^9/L	0.33 (0.27, 0.41)	0.39 (0.32, 0.48)	0.33 (0.30, 0.40)	0.35 (0.29, 0.42) *
MPV, fL	10.60 (10.00, 11.20)	10.45 (9.90, 11.10)	10.45 (10.10, 11.50)	10.60 (10.00, 11.20)
ALB, g/L	48.70 (46.90, 50.50)	49.35 (47.40, 51.20)	49.10 (47.80, 51.00)	48.90 (47.30, 50.70) *
NLR	1.61 (1.22, 2.07)	1.64 (1.32, 2.05)	1.51 (1.30, 1.84)	1.53 (1.20, 1.96) *
d_NLR	1.28 (1.00, 1.62)	1.25 (1.08, 1.57)	1.22 (1.05, 1.50)	1.24 (0.99, 1.57)
PLR	125.63 (100.00, 156.13)	111.59 (88.33, 142.07)	120.92 (109.89, 147.29)	115.85 (94.82, 141.97) *
SII	380.65 (277.99, 525.27)	387.30 (297.08, 522.16)	397.72 (295.95, 486.51)	386.46 (292.44, 517.01)
SIRI	0.52 (0.36, 0.75)	0.65 (0.48, 0.89)	0.49 (0.39, 0.62)	0.54 (0.39, 0.75) *
LMR	5.88 (4.68, 7.32)	5.46 (4.35, 6.68)	6.15 (4.96, 6.93)	6.28 (5.12, 7.68) *
ALI	696.28 (528.05, 916.81)	793.54 (629.4, 1003.11)	693.49 (571.58, 839.64)	842.87 (649.39, 1081.07) *
CA	0.02 (0.01, 0.04)	0.03 (0.02, 0.05)	0.02 (0.01, 0.03)	0.03 (0.02, 0.05) *

Data are presented as median with the interquartile range [M (P25-P75)], or frequency (percentage) [n (%)]

*T2DM* type 2 diabetes mellitus, *WC* waist circumference, *BMI* body mass index, *SBP* systolic blood pressure, *DBP* diastolic pressure, *TG* triglyceride, *HDL-C* high-density lipoprotein cholesterol, *FBG* fasting blood glucose, *2h PG* 2-h post-load blood glucose, *FINs* Fasting insulin, *ALT* alanine aminotransferase, *AST* aspartate aminotransferase, *CRP* C-reactive protein, *WBC* white blood cell, *LYMPH* lymphocyte, *NEUT* neutrophils, *MONO* monocyte, *MPV* mean platelet volume, *ALB* albumin, *NLR* neutrophils-to-lymphocyte ratio, *SII* systemic immune inflammation index, *SIRI* systemic immune inflammation response index

^*^P < 0.05 for the comparison of MAFLD-only, NAFLD-only, and overlap-FLD by Kruskal–Wallis test or Chi-squared test

### Inflammatory status of MAFLD with/without CRP

Considering that CRP was an item in the MAFLD definition, CRP was removed and re-defined MAFLD. Only 10 participants were excluded from the fully defined MAFLD group. No differences in SI indicators were observed after excluding the 10 participants. The Mann–Whitney U test was used to explore the relationship between SI indicators and MAFLD, and no significant differences in SI indicators were found between the re-defined and fully defined MAFLD (Table 4).

**Table 4:** Evaluation of inflammatory status of MAFLD with and without CRP

Indicators	Overall (*n* = 6718)	Non-MAFLD^a^ (*n* = 4388)	MAFLD^a^ (*n* = 2330)	Non-MAFLD^b^ (*n* = 4398)	MAFLD^b^ (*n* = 2320)
CRP, mg/L	1.11 (0.73, 2.04)	1.00 (0.68, 1.74)	1.39 (0.87, 2.50)*	1.00 (0.68, 1.75)	1.39 (0.87, 2.50)^+^
WBC, 10^9/L	5.74 (4.90, 6.78)	5.52 (4.72, 6.55)	6.11 (5.32, 7.11)*	5.52 (4.72, 6.55)	6.11 (5.32, 7.11)^+^
LYMPH, 10^9/L	2.01 (1.65, 2.43)	1.92 (1.58, 2.32)	2.17 (1.81, 2.60)*	1.92 (1.58, 2.32)	2.18 (1.81, 2.61)^+^
NEUT, 10^9/L	3.17 (2.56, 3.96)	3.05 (2.47, 3.83)	3.36 (2.78, 4.14)*	3.05 (2.47, 3.84)	3.36 (2.78, 4.14)^+^
MONO, 10^9/L	0.34 (0.27, 0.41)	0.33 (0.27, 0.40)	0.35 (0.29, 0.43)*	0.33 (0.27, 0.41)	0.35 (0.29, 0.43)^+^
MPV, fL	10.60 (10.00, 11.20)	10.60 (10.00, 11.20)	10.50 (10.00, 11.10)	10.60 (10.00, 11.20)	10.50 (10.00, 11.10)
ALB, g/L	48.80 (47.00, 50.60)	48.70 (46.90, 50.50)	49.00 (47.30, 50.80)*	48.70 (46.90, 50.50)	48.95 (47.30, 50.75)^+^
NLR	1.57 (1.22, 2.04)	1.60 (1.22, 2.07)	1.53 (1.22, 1.96)*	1.60 (1.22, 2.07)	1.53 (1.22, 1.96)^+^
d_NLR	1.27 (1.00, 1.61)	1.28 (1.00, 1.62)	1.24 (1.00, 1.57)	1.28 (1.00, 1.62)	1.24 (1.00, 1.57)^+^
PLR	121.43 (97.95, 150.53)	125.46 (100.00, 156.1)	114.98 (93.91, 142.00)*	125.55 (100.00, 156.10)	114.98 (93.78, 141.97)^+^
SII	382.53 (284.17, 521.14)	380.66 (278.16, 524.12)	386.62 (293.20, 517.52)	380.67 (278.21, 524.78)	386.46 (292.91, 517.18)
SIRI	0.53 (0.37, 0.75)	0.52 (0.36, 0.75)	0.55 (0.39, 0.76)*	0.52 (0.36, 0.75)	0.55 (0.39, 0.76)^+^
LMR	5.99 (4.78, 7.43)	5.88 (4.68, 7.31)	6.21 (5.05, 7.57)*	5.88 (4.68, 7.30)	6.21 (5.05, 7.57)^+^
ALI	740.39 (562.69, 974.88)	696.1 (528.42, 915.45)	839.78 (646.5, 1072.40)*	696.01 (528.37, 915.36)	840.86 (648.4, 1073.14)^+^
CA	0.02 (0.01, 0.04)	0.02 (0.01, 0.04)	0.03 (0.02, 0.05)*	0.02 (0.01, 0.04)	0.03 (0.02, 0.05)^+^

Data are presented as median with the interquartile range [M (P25-P75)]

*CRP* C-reactive protein, *WBC* white blood cell, *LYMPH* lymphocyte, *NEUT* neutrophils, *MONO* monocyte, *MPV* mean platelet volume, *ALB* albumin, *NLR* neutrophils-to-lymphocyte ratio, *SII* systemic immune inflammation index, *SIRI* systemic immune inflammation response index

^a^MAFLD fully defined according to the standard

^b^MAFLD defined after removing CRP from diagnostic criteria

^*^P < 0.05 for MAFLD and non-MAFLD according to the standard by Wilcoxon Signed Rank test

^+^*P* < 0.05 for the comparison of MAFLD and non-MAFLD after removing CRP by Wilcoxon Signed Rank test

### Relationship between SI indicators and MAFLD/NAFLD

**Fig. 2:**
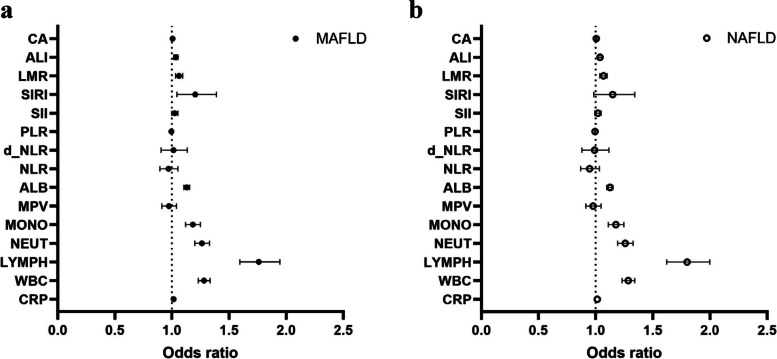
Logistic regression analysis of the relationship between systemic inflammatory indicators and MAFLD **a** and NAFLD **b**

Logistic regression analyses were used to explore the relationship between SI indicators and MAFLD, and the results are shown in Fig. 2 (a) and Supplementary Table 1. Except for MPV and SII, the ORs of other SI indicators were statistically significant in crude models. After adjusting for age, sex, BMI, smoking history, alcohol drinking history, education, and occupation, CRP, WBC, LYMPH, NEUT, MONO, ALB, PLR, SIRI, LMR, ALI, and CA were positively associated with MAFLD prevalence, and PLR was negatively associated. RCS analysis showed that a linear relationship existed between MPV, ALB, NLR, d_NLR, and PLR and MAFLD, whereas CRP, WBC, LYMPH, NEUT, MONO, SII, SIRI, LMR, ALI, and CA exhibited a non-linear relationship with MAFLD (Supplementary Fig. 1).

The results for the relationship between SI indicators and NAFLD are shown in Fig. 2 (b) and Supplementary table 2. The ORs of CRP, WBC, LYMPH, NEUT, MONO, ALB, PLR, LMR, ALI, and CA were greater than 1.0, and the OR of PLR was less than 1.0 in multivariable-adjusted logistic regression analysis. RCS analysis showed that there was a linear relationship between MPV, ALB, NLR, d_NLR, PLR, SIRI, and LMR and NAFLD, whereas CRP, WBC, LYMPH, NEUT, MONO, SII, ALI, and CA showed a non-linear relationship with NAFLD (Supplementary Fig. 2).

### ROC analysis of SI indicators in MAFLD and NAFLD

**Fig. 3:**
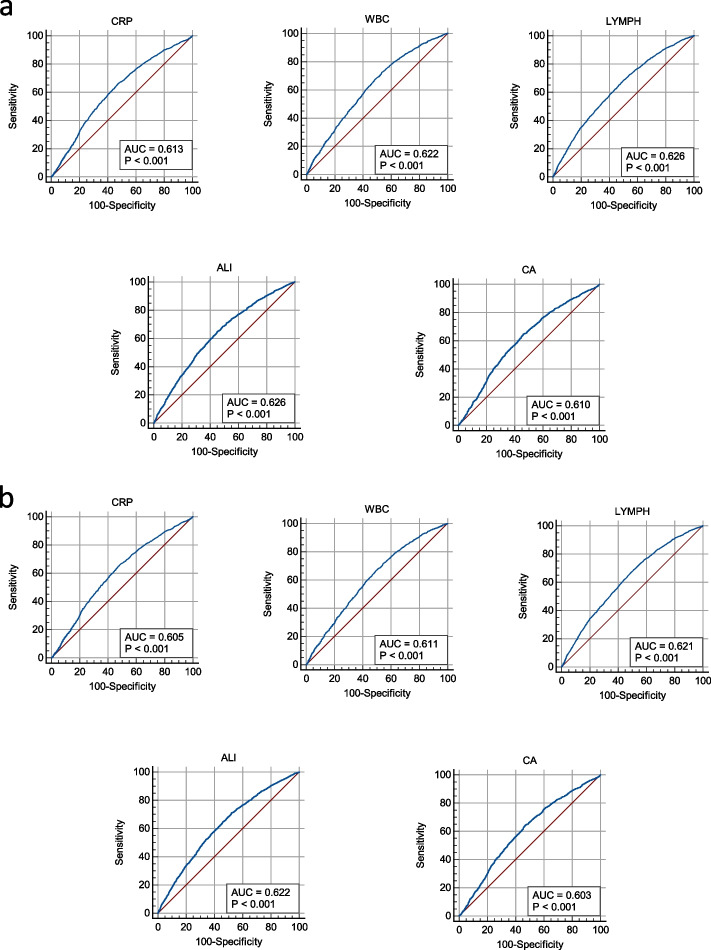
Diagnostic accuracy of systemic inflammatory indicators for MAFLD **a** and NAFLD **b**

The AUC, sensitivity, specificity, and positive predictive values of SI indicators for MAFLD and NAFLD are depicted in Fig. 3, Supplementary table 3 and Supplementary Figs. 3 and  4. The AUC values of all SI indicators were lower than 0.7 in both MAFLD and NAFLD. The AUC values of CRP, WBC, LYMPH, ALI and CA were all higher than 0.60 for both MAFLD (0.61, 0.62, 0.63, 0.63 and 0.60 respectively) and NAFLD (0.61, 0.61, 0.62, 0.62 and 0.60 respectively), and their ROCs are presented in Supplementary Fig. 5. The AUCs of MPV and SII were lower than those of other indicators for both MAFLD (0.51 and 0.51, respectively) and NAFLD (0.50 and 0.51, respectively). The sensitivity, specificity, and positive predictive values of LYMPH, ALI, and MPV in MAFLD were 0.69, 0.50, and 0.42, 0.65, 0.55, and 0.43, and 0.15, 0.82, and 0.31, respectively. The sensitivity, specificity, and positive predictive values of LYMPH, ALI, and MPV in NAFLD were 0.69, 0.49, and 0.38, 0.71, 0.47, and 0.38, and 0.86, 0.16, and 0.32, respectively.

## Discussion

The current study compared the prevalence and SI levels of MAFLD and NAFLD in a general population. The prevalence of MAFLD was 34.7%, slightly higher than 32.4% of NAFLD. Their overlapping rate was 89.7%, while only 8.3% and 1.9% of participants were MAFLD-only and NAFLD-only, respectively. Of the 15 SI indicators, 12 indicators showed striking differences between MAFLD and non-MAFLD, and between NAFLD and non-NAFLD. Moreover, the MAFLD-only population showed slightly higher SI levels than the overlap-FLD group. Both MAFLD-only and overlap-FLD groups had a worse SI status than the NAFLD-only group. The results were similar after removing CRP from the definition of MAFLD. Among all the SI indicators, LYMPH and ALI were closely associated with MAFLD and NAFLD. However, they still showed poor discrimination ability between MAFLD and non-MAFLD as well as NAFLD and non-NAFLD.

NAFLD is closely associated with the presence and severity of multiple metabolic disorders [33]. With the developing understanding of the mechanism of NAFLD, the nomenclature of NAFLD has changed to MAFLD, underscoring the underlying pathophysiology of NAFLD as a metabolically driven disease [4, 34]. A recent meta-analysis showed that the prevalence of MAFLD and NAFLD was 39.22% and 38.28%, respectively, and MAFLD identified more FLD than NAFLD [7]. In China, both higher and lower prevalence rates of MAFLD than NAFLD have been reported [13, 35]. In the current study, FLD was diagnosed in the general population by strictly trained professional sonographers using two similar color ultrasonic scanners with a color Doppler ultrasound system, and the prevalence of MAFLD and NAFLD was 34.8% and 31.3%, respectively.

From the definition, NAFLD mainly focuses on the exclusion of hepatic steatosis driven by competing etiologies, such as alcohol, hepatitis B, etc., while MAFLD relies more on associated comorbidities [34]. After the population was reorganized, the MAFLD-only, NAFLD-only, and overlap-FLD groups were composed of 8.3%, 1.9%, and 89.7% of FLD patients, respectively. Compared with the overlap-FLD group, the MAFLD-only group showed higher blood glucose, BP, and blood lipids, whereas the NAFLD-only group had fewer metabolic disorders. These suggested that MAFLD could cover more FLD regardless of alcohol drinking, and ruled out metabolic healthier FLD.

The results of this study are consistent with the findings of a meta-analysis involving 17 studies, which demonstrated that of 9,808,677 FLD patients, 15.1% were MAFLD-only, 4.0% were NAFLD-only, and 80.9% met the criteria of MAFLD and NAFLD simultaneously; the MAFLD-only group had a higher risk for abnormal liver function and fibrosis [6]. Overall, these data suggest that MAFLD could better identify individuals with adverse metabolic status, with/without the presence of secondary causes for steatosis.

Inflammation plays an important role in the development and progression of NAFLD [21]. However, only a few studies have reported the inflammation levels of MAFLD, and no study has explored the difference in the SI status between MAFLD and NAFLD. Thus, all previous reports assessing the SI status were reviewed, and 15 SI indicators were included in the current study. Both MAFLD and NAFLD were positively associated with higher levels of SI indicators. Meanwhile, MAFLD-only and overlap-FLD groups had higher inflammation levels than NAFLD-only. The NAFLD-only population showed slightly higher inflammation levels than the non-FLD group. This result was similar to previous findings on liver enzymes, in which MAFLD was associated with higher levels of ALT and AST and a higher risk for fibrosis compared with NAFLD, and MAFLD was superior to NAFLD in identifying adverse liver outcomes [6]. Furthermore, one study has demonstrated that MAFLD may offer better identification of atherosclerotic cardiovascular disease (ASCVD) risk compared to NAFLD [36] Another study suggested that MAFLD might be more effective in identifying subjects at risk of cardiovascular disease (CVD) than NAFLD, and that fibrosis assessment could further refine prognostication in subjects with MAFLD [37]. Given that inflammation plays a pivotal role in disease progression and fibrosis development [38, 39], Therefore, it is worthwhile to delve deeper into whether the differential inflammatory factors between MAFLD and NAFLD contribute significantly to the better identification of individuals at risk of CVD. Additionally, other studies have reported associations between MAFLD and Psoriasis through IL-17 in hepatocytes mediating systemic inflammation and mobilization of inflammatory cells to the liver [40]. It has also been suggested that MAFLD could influence psoriasis severity by releasing inflammatory mediators from hepatocytes, including reactive oxygen species, C-reactive protein and interleukin-6 [40]. Inflammation—as both a driver and response of liver damage [41]—may help explain this phenomenon. Taken together, these findings suggest that MAFLD could capture more severe inflammation status than NAFLD. Hence, identifying inflammatory factors capable of discerning MAFLD holds potential for novel therapeutic approaches for patients with this condition.

CRP, a commonly used marker of low-grade inflammation, was a component in MAFLD definition, and it may lead to a false positive result when analyzing the SI status of MAFLD/NAFLD and their subgroups. Thus, CRP was excluded and redefined MAFLD. Only 10 participants (0.4%) were excluded from fully-defined MAFLD. Comparisons between MAFLD and non-MAFLD groups or among MAFLD-only, NAFLD-only, and overlap-FLD groups showed no marked change in their SI indicators. While the impact of CRP on the MAFLD definition was deemed modest in this study, it is worth noting that CRP exhibited significant differences between groups. In a prior investigation, Tsubasa Tsutsumi et al. proposed that CRP/albumin ratio could potentially be a crucial factor influencing COPD morbidity in MAFLD patients [42]. Therefore, further investigation is warranted to explore the potential value of CRP in the clinical diagnosis of MAFLD.

Finally, the diagnostic value of all SI indicators in MAFLD/NAFLD diagnosis were evaluated. Both logistic regression and RCS analyses showed that CRP, WBC, MONO, and LMR were positively associated with the prevalence of MAFLD and NAFLD, while PLR showed an inverse association, which is consistent with previous studies [43–46]. In the ROC analysis, the AUC values of CRP, WBC, LYMPH, ALI, and CA ranged from 0.60 to 0.63 in both MAFLD and NAFLD prediction, and the AUC values of other SI indicators were all less than 0.60. Of these SI indicators, CRP had the best predictive potential, but still with low predictive power. However, only indicators clinically highly accessible and generally representing inflammation were included in the current study, and thus more specific inflammatory indicators, such as interleukin, tumor necrosis factor, etc., should be examined in further studies, to explore the inflammation characteristic with the transformation from NAFLD to MAFLD.

### Strengths and limitations

The main strength of this study is a relatively large-scale general population-based study, with FLD diagnosed using ultrasound and a comprehensive measurement of metabolic parameters with strict study settings and good quality control. Additionally, the SI indicators of MAFLD and NAFLD were summarized, and the subgroups were regrouped into MAFLD-only, NAFLD-only, and overlap-FLD groups, showing a comprehensive SI status from NAFLD to MALFD. Nevertheless, this study has some limitations. First, an ultrasonographic examination was used in the diagnosis of FLD instead of liver biopsy — the gold standard method — as it is non-invasive and more acceptable and feasible in large population-based studies. Second, the study population was less representative because it included local participants aged 35–74 years, with more females. Third, only reported SI indicators easy-to-detect in clinical settings were included, and therefore more specific inflammation cytokines should be examined. Fourth, besides alcohol consumption, other factors should be considered in the diagnose of NAFLD, such as positive hepatitis B surface antigen, antibody against hepatitis C virus; autoimmune liver, competing etiologies of liver disease resulting in steatosis, and so on. However, they were not checked systemically in the study population, thus may lead to the mistakes in NAFLD diagnose. Finally, although this cross-sectional study pointed out a plausible difference in SI levels between NAFLD and MAFLD, the causal inference was not feasible, thus future cohort studies are needed.

## Conclusion

In summary, the current study found that the prevalence rates of MAFLD and NAFLD were 34.7% and 32.4%, respectively, in Southern China. The overlapping rate was 89.7%. MAFLD-only and NAFLD-only groups consisted of 8.3% and 1.9%, respectively, of all FLD. Both MAFLD and NAFLD were closely associated with a higher SI status than non-patients. The MAFLD-only group had a more severe inflammation status, while the NAFLD-only group exhibited lower levels. Therefore, the MAFLD criteria may be more appropriate in population studies in terms of inflammatory status, which will allow for early lifestyle or medical interventions and prevent further disease progression. However, whether it is necessary to include CRP in the MAFLD definition should be reconsidered. Moreover, the findings of this paper should be further validated to determine the long-term effect of MAFLD.

### Supplementary Information

**Additional file 1:** **Supplementary Table 1.** Logistic regression analysis of the relationship between systemic inflammatory indicators and MAFLD. **Supplementary Table 2.** Logistic regression analysis of the relationship between systemic inflammatory indicators and NAFLD. **Supplementary Table 3.** Diagnostic accuracy of systemic inflammatory indicators for MAFLD and NAFLD. **Supplementary Figure 1.** RCS analysis of systemic inflammatory indicators and MAFLD. **Supplementary Figure 2.** RCS analysis of systemic inflammatory indicators and NAFLD. **Supplementary Figure 3.** Diagnostic accuracy of systemic inflammatory indicators for MAFLD. **Supplementary Figure 4.** Diagnostic accuracy of systemic inflammatory indicators for NAFLD. **Supplementary Figure 5.** Comparison of multiple indicators ROC for MAFLD (a) and NAFLD (b).

## Data Availability

The data that support the findings of this study are not openly available due to reasons of sensitivity and are available from the corresponding author upon reasonable request.

## Abbreviations

NAFLD: Nonalcoholic fatty liver disease
MAFLD: Metabolic dysfunction-associated fatty liver disease
SBP: Systolic blood pressure
DBP: Diastolic blood pressure
BMI: Body mass index
WC: Waist circumstance
OGTT: Oral glucose tolerance test
FBG: Fasting blood glucose
2 h PG: 2-H post-load blood glucose
TG: Triglyceride
HDL-c: High-density lipoprotein cholesterol
ALB: Albumin
CRP: C-reactive protein
HbA1c: Glycosylated hemoglobin
FINs: Fasting insulin
HOMA-IR: Homeostasis model assessment of insulin resistance
WBC: White blood cell
LYMPH: Lymphocyte
NEUT: Neutrophils
MONO: Monocyte
PLT: Platelet
MPV: Mean platelet volume
T2DM: Type 2 diabetes mellitus
NLR: Neutrophils-to-lymphocyte ratio
SII: Systemic immune inflammation index
SIRI: Systemic immune inflammation response index
