# Supplementary material for: Shift in prevalence and systemic inflammation levels from NAFLD to MAFLD: a population-based cross-sectional study
Source: Lipids Health Dis. 2023 Oct 28;22:185. doi: 10.1186/s12944-023-01947-4 (PMC10613356; doi:10.1186/s12944-023-01947-4)
Supplement: Supplementary file 1 — Additional file 1: Supplementary Table 1. Logistic regression analysis of the relationship between systemic inflammatory indicators and MAFLD. Supplementary Table 2. Logistic regression analysis of the relationship between systemic inflammatory indicators and NAFLD. Supplementary Table 3. Diagnostic accuracy of systemic inflammatory indicators for MAFLD and NAFLD. Supplementary Figure 1. RCS analysis of systemic inflammatory indicators and MAFLD. Supplementary Figure 2. RCS analysis of systemic inflammatory indicators and NAFLD. Supplementary Figure 3. Diagnostic accuracy of systemic inflammatory indicators for MAFLD. Supplementary Figure 4. Diagnostic accuracy of systemic inflammatory indicators for NAFLD. Supplementary Figure 5. Comparison of multiple indicators ROC for MAFLD (a) and NAFLD (b). [file 12944_2023_1947_MOESM1_ESM.docx]

| **Supplementary Table 1. Logistic regression analysis of the relationship between systemic inflammatory indicators and MAFLD** | | | | | | | | | |
| --- | --- | --- | --- | --- | --- | --- | --- | --- | --- |
| Indicators |  | Model 1 | |  | Model 2 | |  | Model 3 | |
|  |  | OR (95CI%) | *P* |  | OR (95CI%) | *P* |  | OR (95CI%) | *P* |
| CRP, mg/L |  | 1.026 (1.014, 1.039) | 0.005 |  | 1.024 (1.012, 1.036) | <0.001 |  | 1.015 (1.004, 1.027) | 0.010 |
| WBC, 10^9/L |  | 1.295 (1.251, 1.339) | <0.001 |  | 1.302 (1.257, 1.348) | <0.001 |  | 1.281 (1.231, 1.334) | <0.001 |
| LYMPH, 10^9/L |  | 1.986 (1.822, 2.165) | <0.001 |  | 1.968 (1.805, 2.146) | <0.001 |  | 1.760 (1.594, 1.944) | <0.001 |
| NEUT, 10^9/L |  | 1.250 (1.198, 1.304) | <0.001 |  | 1.255 (1.201, 1.311) | <0.001 |  | 1.263 (1.200, 1.329) | <0.001 |
| MONO, 10^9/L |  | 1.195 (1.144, 1.248) | <0.001 |  | 1.210 (1.156, 1.267) | <0.001 |  | 1.183 (1.120, 1.249) | <0.001 |
| MPV, fL |  | 0.966 (0.914, 1.022) | 0.234 |  | 0.980 (0.926, 1.037) | 0.486 |  | 0.974 (0.912, 1.041) | 0.437 |
| ALB, g/L |  | 1.045 (1.026, 1.064) | <0.001 |  | 1.060 (1.040, 1.080) | <0.001 |  | 1.130 (1.105, 1.156) | <0.001 |
| NLR |  | 0.902 (0.841, 0.967) | 0.004 |  | 0.898 (0.837, 0.964) | 0.003 |  | 0.970 (0.895, 1.052) | 0.464 |
| d_NLR |  | 0.905 (0.821, 0.997) | 0.043 |  | 0.905 (0.821, 0.998) | 0.045 |  | 1.014 (0.906, 1.135) | 0.803 |
| PLR |  | 0.994 (0.993, 0.995) | <0.001 |  | 0.994 (0.993, 0.995) | <0.001 |  | 0.996 (0.994, 0.997) | <0.001 |
| SII |  | 1.067 (1.042, 1.093) | 0.944 |  | 1.004 (0.982, 1.026) | 0.745 |  | 1.025 (0.999, 1.052) | 0.059 |
| SIRI |  | 1.001 (0.979, 1.023) | 0.039 |  | 1.122 (0.991, 1.269) | 0.068 |  | 1.204 (1.044, 1.389) | 0.011 |
| LMR |  | 1.133 (1.006, 1.277) | <0.001 |  | 1.074 (1.048, 1.102) | <0.001 |  | 1.063 (1.032, 1.095) | <0.001 |
| ALI |  | 1.124 (1.106, 1.141) | <0.001 |  | 1.126 (1.108, 1.143) | <0.001 |  | 1.035 (1.017, 1.053) | <0.001 |
| CA |  | 1.011 (1.006, 1.017) | 0.013 |  | 1.010 (1.005, 1.016) | <0.001 |  | 1.006 (1.001, 1.012) | 0.024 |
| Note: Model 1: Unadjusted. Model 2: Adjusted for age and sex. Model 3: Adjusted for age, sex, BMI, smoking history, drinking history, educational level and occupation. CRP: C-reactive protein; WBC: white blood cell; LYMPH: lymphocyte; NEUT: neutrophils; MONO: monocyte; MPV: mean platelet volume; ALB: albumin; NLR: neutrophils-to-lymphocyte ratio; SII: systemic immune inflammation index; SIRI: systemic immune inflammation response index. | | | | | | | | | |

| **Supplementary Table 2. Logistic regression analysis of the relationship between systemic inflammatory indicators and NAFLD** | | | | | | | | | |
| --- | --- | --- | --- | --- | --- | --- | --- | --- | --- |
| Indicators |  | Model 1 | |  | Model 2 | |  | Model 3 | |
|  |  | OR (95CI%) | *P* |  | OR (95CI%) | *P* |  | OR (95CI%) | *P* |
| CRP, mg/L |  | 1.019 (1.008, 1.030) | 0.001 |  | 1.018 (1.007, 1.029) | 0.001 |  | 1.015 (1.002, 1.027) | 0.021 |
| WBC, 10^9/L |  | 1.244 (1.203, 1.287) | <0.001 |  | 1.280 (1.236, 1.326) | <0.001 |  | 1.285 (1.231, 1.341) | <0.001 |
| LYMPH, 10^9/L |  | 1.888 (1.731, 2.059) | <0.001 |  | 1.899 (1.740, 2.073) | <0.001 |  | 1.799 (1.621, 1.997) | <0.001 |
| NEUT, 10^9/L |  | 1.200 (1.150, 1.253) | <0.001 |  | 1.236 (1.183, 1.292) | <0.001 |  | 1.258 (1.192, 1.327) | <0.001 |
| MONO, 10^9/L |  | 1.110 (1.062, 1.160) | <0.001 |  | 1.179 (1.124, 1.236) | <0.001 |  | 1.177 (1.111, 1.247) | <0.001 |
| MPV, fL |  | 0.997 (0.941, 1.056) | 0.923 |  | 1.001 (0.945, 1.061) | 0.973 |  | 0.978 (0.914, 1.047) | 0.527 |
| ALB, g/L |  | 1.039 (1.020, 1.059) | <0.001 |  | 1.057 (1.037, 1.078) | <0.001 |  | 1.125 (1.098, 1.151) | <0.001 |
| NLR |  | 0.875 (0.813, 0.940) | <0.001 |  | 0.902 (0.838, 0.970) | 0.006 |  | 0.948 (0.870, 1.033) | 0.226 |
| d_NLR |  | 0.886 (0.801, 0.979) | 0.018 |  | 0.915 (0.827, 1.012) | 0.084 |  | 0.991 (0.880, 1.116) | 0.880 |
| PLR |  | 0.995 (0.993, 0.996) | <0.001 |  | 0.994 (0.993, 0.996) | <0.001 |  | 0.995 (0.994, 0.997) | <0.001 |
| SII |  | 1.095 (1.068, 1.122) | 0.814 |  | 1.003 (0.980, 1.026) | 0.800 |  | 1.020 (0.993, 1.048) | 0.154 |
| SIRI |  | 0.997 (0.975, 1.020) | 0.739 |  | 1.077 (0.948, 1.225) | 0.254 |  | 1.149 (0.984, 1.341) | 0.079 |
| LMR |  | 0.979 (0.864, 1.110) | <0.001 |  | 1.077 (1.050, 1.105) | <0.001 |  | 1.069 (1.037, 1.101) | <0.001 |
| ALI |  | 1.115 (1.098, 1.133) | <0.001 |  | 1.110 (1.093, 1.127) | <0.001 |  | 1.038 (1.019, 1.057) | <0.001 |
| CA |  | 1.008 (1.003, 1.013) | 0.001 |  | 1.008 (1.003, 1.013) | 0.003 |  | 1.006 (1.000, 1.012) | 0.042 |
| Note: Model 1: Unadjusted. Model 2: Adjusted for age and sex. Model 3: Adjusted for age, sex, BMI, smoking history, drinking history, educational level and occupation. CRP: C-reactive protein; WBC: white blood cell; LYMPH: lymphocyte; NEUT: neutrophils; MONO: monocyte; MPV: mean platelet volume; ALB: albumin; NLR: neutrophils-to-lymphocyte ratio; SII: systemic immune inflammation index; SIRI: systemic immune inflammation response index. | | | | | | | | | |

| **Supplementary Table 3. Diagnostic accuracy of systemic inflammatory indicators for MAFLD and NAFLD** | | | | | | | | | | | |
| --- | --- | --- | --- | --- | --- | --- | --- | --- | --- | --- | --- |
| Indicators | MAFLD | | | | |  | NAFLD | | | | |
|  | cut-off | AUC (95%*CI*) | sensitivity | specificity | positive_pre |  | cut-off | AUC (95%*CI*) | sensitivity | specificity | positive_pre |
| CRP, mg/L | 1.045 | 0.61 (0.60, 0.62) | 0.658 | 0.528 | 0.425 |  | 1.035 | 0.61 (0.60, 0.62) | 0.656 | 0.512 | 0.380 |
| WBC, 10^9/L | 5.045 | 0.62 (0.61, 0.63) | 0.728 | 0.460 | 0.399 |  | 5.565 | 0.61 (0.60, 0.63) | 0.668 | 0.504 | 0.379 |
| LYMPH, 10^9/L | 1.915 | 0.63 (0.61, 0.64) | 0.688 | 0.495 | 0.420 |  | 1.915 | 0.62 (0.61, 0.64) | 0.691 | 0.490 | 0.380 |
| NEUT, 10^9/L | 3.025 | 0.59 (0.58, 0.60) | 0.651 | 0.491 | 0.405 |  | 2.935 | 0.57 (0.44, 0.68) | 0.681 | 0.443 | 0.357 |
| MONO, 10^9/L | 0.315 | 0.57 (0.57, 0.58) | 0.659 | 0.459 | 0.392 |  | 0.315 | 0.58 (0.56, 0.59) | 0.644 | 0.482 | 0.346 |
| MPV, fL | 11.450 | 0.51 (0.50, 0.52) | 0.154 | 0.818 | 0.310 |  | 9.750 | 0.50 (0.49, 0.52) | 0.841 | 0.179 | 0.317 |
| ALB, g/L | 47.450 | 0.53 (0.52, 0.56) | 0.733 | 0.328 | 0.366 |  | 47.450 | 0.53 (0.51, 0.54) | 0.733 | 0.326 | 0.331 |
| NLR | 1.992 | 0.52 (0.51, 0.53) | 0.530 | 0.523 | 0.363 |  | 1.885 | 0.53 (0.51, 0.54) | 0.529 | 0.534 | 0.332 |
| d_NLR | 1.271 | 0.51 (0.50, 0.53) | 0.535 | 0.512 | 0.368 |  | 1.269 | 0.52 (0.50, 0.53) | 0.537 | 0.513 | 0.334 |
| PLR | 125.831 | 0.57 (0.56, 0.58) | 0.615 | 0.497 | 0.393 |  | 125.042 | 0.56 (0.55, 0.58) | 0.612 | 0.493 | 0.355 |
| SII | 288.913 | 0.51 (0.50, 0.52) | 0.763 | 0.274 | 0.358 |  | 323.361 | 0.51 (0.50, 0.51) | 0.677 | 0.357 | 0.326 |
| SIRI | 0.372 | 0.53 (0.51, 0.54) | 0.758 | 0.290 | 0.361 |  | 0.372 | 0.51 (0.50, 0.53) | 0.772 | 0.262 | 0.322 |
| LMR | 5.342 | 0.55 (0.53, 0.56) | 0.690 | 0.397 | 0.378 |  | 5.345 | 0.56 (0.55, 0.58) | 0.737 | 0.368 | 0.350 |
| ALI | 724.936 | 0.63 (0.61, 0.64) | 0.650 | 0.549 | 0.433 |  | 682.528 | 0.62 (0.61, 0.64) | 0.712 | 0.477 | 0.382 |
| CA | 0.022 | 0.60 (0.61, 0.62) | 0.647 | 0.538 | 0.427 |  | 0.022 | 0.60 (0.59, 0.62) | 0.644 | 0.530 | 0.381 |
| Note: CRP: C-reactive protein; WBC: white blood cell; LYMPH: lymphocyte; NEUT: neutrophils; MONO: monocyte; MPV: mean platelet volume; ALB: albumin; NLR: neutrophils-to-lymphocyte ratio; SII: systemic immune inflammation index; SIRI: systemic immune inflammation response index. | | | | | | | | | | | |


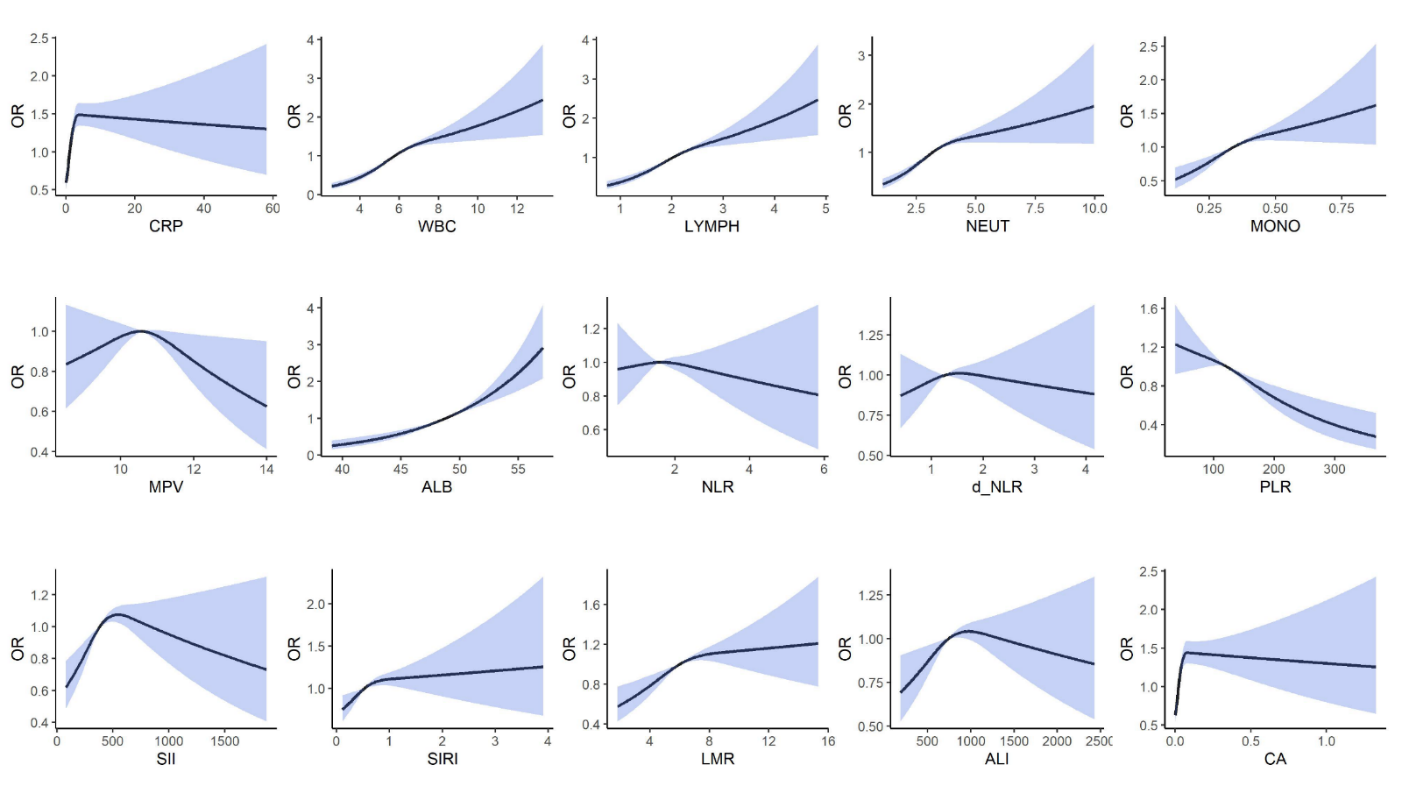


**Supplementary Figure 1. RCS analysis of systemic inflammatory indicators and MAFLD**


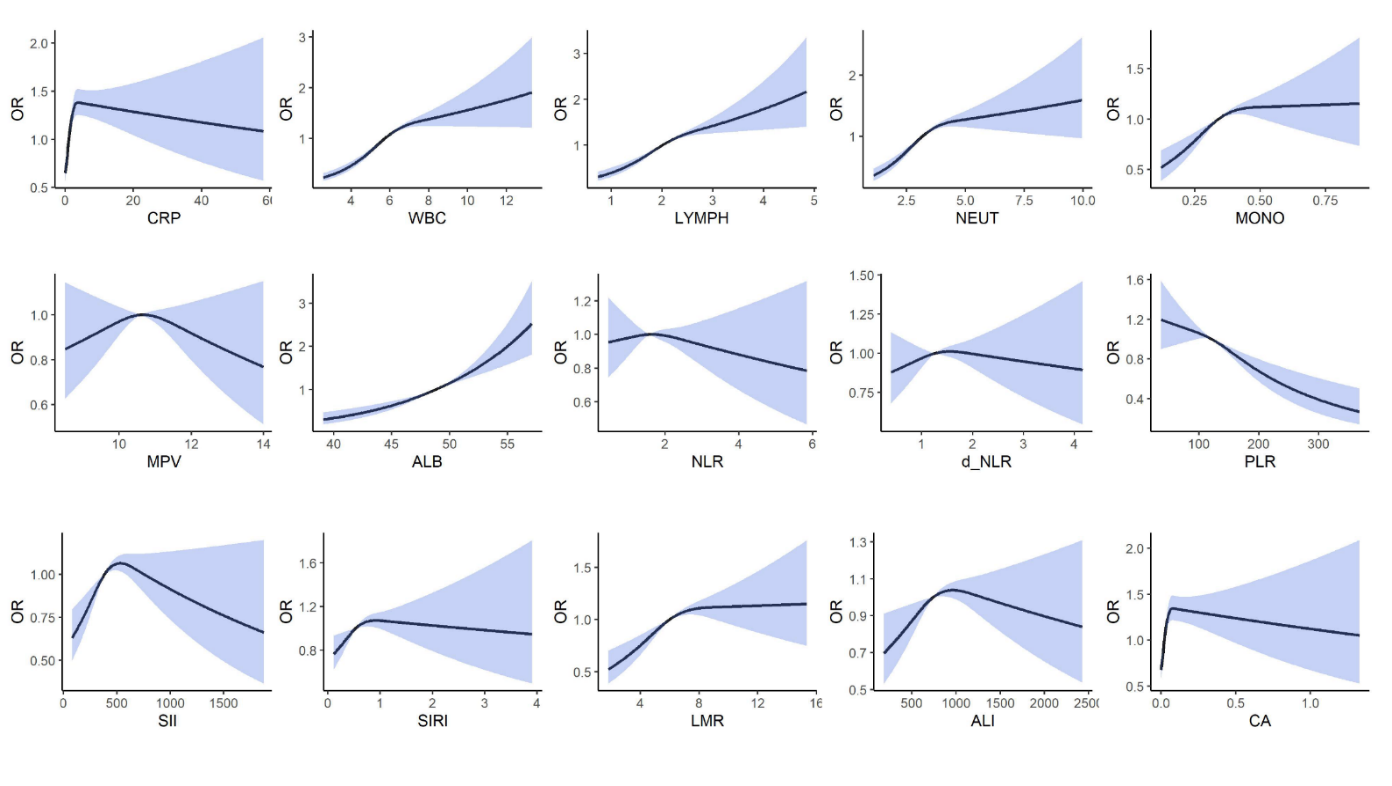


**Supplementary Figure 2. RCS analysis of systemic inflammatory indicators and NAFLD**

**Supplementary Figure 3. Diagnostic accuracy of systemic inflammatory indicators for MAFLD.**

**Supplementary Figure 4. Diagnostic accuracy of systemic inflammatory indicators for NAFLD.**

b

a

**Supplementary Figure 5. Comparison of multiple indicators ROC for MAFLD (a) and NAFLD (b).**
